# 

**Published:** 1881-09

**Authors:** 


CONTRIBUTORS:

Drs. H. C. Allen, T. F. Allen, Edward Bayard, J. B. Bell, E. W. Berridge, Geo. H. Clark, A. C. Cowperthwaite, Ad. Fellger, Wm. J. Guernsey, W. A. Hawley, John Hall, W. M. James, W. H. Jenny, Ad. Lippe, C. Lippe, Malcolm MacFarlan, Thos. Moore, C. F. Nichols, C. Pearson, T. F. Pomeroy, Leila A. Rendell, C. Carleton Smith, P. P. Wells, Wm. P. Wesselhoeft, David Wilson, (London), T. P. Wilson, and many others.

NOTICE.

***All articles for publication, books for review, business communications, etc., must be sent to Editor, 2109 Chestnut Street, Philadelphia.***

PHILADELPHIA:

N. B.—Authors of accepted papers are furnished with copies of the number in which their articles appear; application for such copies to be made when sending papers. Any irregularity in the receipt of this Journal should be promptly reported to Editor.

A prize of fifty dollars will be awarded to author of the best essay printed in this Journal for the year 1881. To be adjudged by three physicians of note.

